# The Histamine H3 Receptor Antagonist E159 Reverses Memory Deficits Induced by Dizocilpine in Passive Avoidance and Novel Object Recognition Paradigm in Rats

**DOI:** 10.3389/fphar.2017.00709

**Published:** 2017-10-12

**Authors:** Alaa Alachkar, Dorota Łażewska, Katarzyna Kieć-Kononowicz, Bassem Sadek

**Affiliations:** ^1^Department of Pharmacology and Therapeutics, College of Medicine and Health Sciences, United Arab Emirates University, Al Ain, United Arab Emirates; ^2^Department of Technology and Biotechnology of Drugs, Faculty of Pharmacy, Jagiellonian University Medical College, Kraków, Poland

**Keywords:** histamine H3 receptor, antagonist, learning, memory impairment, passive avoidance paradigm, novel object recognition, elevated plus maze

## Abstract

The involvement of histamine H3 receptors (H3Rs) in memory is well known, and the potential of H3R antagonists in therapeutic management of neuropsychiatric diseases, e.g., Alzheimer disease (AD) is well established. Therefore, the effects of histamine H3 receptor (H3R) antagonist E159 (2.5–10 mg/kg, i.p.) in adult male rats on dizocilpine (DIZ)-induced memory deficits were studied in passive avoidance paradigm (PAP) and in novel object recognition (NOR) using pitolisant (PIT) and donepezil (DOZ) as standard drugs. Upon acute systemic pretreatment of E159 at three different doses, namely 2.5, 5, and 10 mg/kg, i.p., 2.5 and 5 but not 10 mg/kg of E159 counteracted the DIZ (0.1 mg)-induced memory deficits, and this E159 (2.5 mg)-elicited memory-improving effects in DIZ-induced amnesic model were moderately abrogated after acute systemic administration of scopolamine (SCO), H2R antagonist zolantidine (ZOL), but not with H1R antagonist pyrilamine to the animals. Moreover, the observed memory-enhancing effects of E159 (2.5 mg/kg, i.p.) were strongly abrogated when animals were administered with a combination of SCO and ZOL. Furthermore, the E159 (2.5 mg)-provided significant memory-improving effect of in DIZ-induced short-term memory (STM) impairment in NOR was comparable to the DOZ-provided memory-enhancing effect, and was abolished when animals were injected with the CNS-penetrant histamine H3R agonist *R*-(α)-methylhistamine (RAMH). However, E159 at a dose of 2.5 mg/kg failed to exhibit procognitive effect on DIZ-induced long-term memory (LTM) in NOR. Furthermore, the results observed revealed that E159 (2.5 mg/kg) did not alter anxiety levels and locomotor activity of animals naive to elevated-plus maze (EPM), demonstrating that improved performances with E159 (2.5 mg/kg) in PAP or NOR are unrelated to changes in emotional responding or in spontaneous locomotor activity. These results provide evidence for the potential of drugs targeting H3Rs for the treatment of neuropsychiatric disorders, e.g., AD.

## Introduction

**FIGURE 1:**
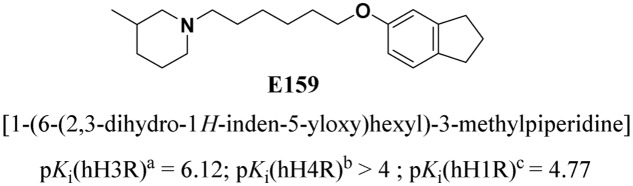
Chemical structure and *in vitro* affinities of the non-imidazole H3R antagonist/inverse agonist E159. ^a^ [^125^I]Iodoproxyfan binding assay at human H3Rstably expressed in CHO-K1 cells, *n* = 3 (Ligneau et al., 1994; Ligneau et al., 2000; Lazewska et al., 2006). ^b^ [^3^H]Histamine binding assay performed with cell membrane preparation of Sf9 cells transiently expressing the human histamine H4Rand co-expressed with G_αi2_ and Gβ1γ2 subunits, *n* = 3 (Meier et al., 2001; Amon et al., 2007; Isensee et al., 2009; Tomasch et al., 2013). ^c^ [^3^H]Pyrilamine binding assay performed with cell membrane preparation of CHO-hH1Rcells stably expressing the human H1R, *n* = 3 (Schibli and Schubiger, 2002; van Staveren and Metzler-Nolte, 2004; Schlotter et al., 2005).

The main representative character of AD as a neurogenerative disease and related dementias, e.g., cognitive deficit associated with schizophrenia (CDS), is the progressive decline in cognitive performance (Medhurst et al., 2007, 2009; Silva et al., 2014), and enhancing cognitive functions in these conditions embodies a multifaceted task, given the fact that various brain neurotransmission systems and several brain regions are involved in the progress of these conditions (Khan et al., 2015; Shimizu et al., 2015; Sadek et al., 2016a,c). Current pharmacological interventions for AD, such as cholinesterase inhibitors, provide only shortly timed marginal clinical benefit (Silva et al., 2014). Hence, the difficulties to develop satisfactory therapies of AD and CDS are still restricted due to the complicated pathophysiology of these diseases including several pathways, e.g., defective β-amyloid protein metabolism and abnormalities in central neurotransmissions for acetylcholine, glutamate, noradrenaline, serotonin, and dopamine, and the association of these diseases with inflammatory and/or oxidative and hormonal pathways (Doraiswamy, 2002; Cavalli et al., 2008; Zhou et al., 2016). Importantly, the brain histaminergic system’s role in AD has been proposed, and a variety of pharmaceutical agents targeting central histaminergic systems have been developed (Bishara, 2010; Baronio et al., 2014; Sadek and Stark, 2015; Sadek et al., 2016c). Accordingly, H3Rs functioning as auto-receptors modulate synthesis and release of central histamine (Sadek and Stark, 2015; Sadek et al., 2016c). Moreover, dysregulations in a wide range of different central neurotransmitter systems, e.g., dopamine, serotonin, GABA, and glutamate were generally hypothesized for the H3Rs located on neurons other than histmainergic neural cells and functioning as hetero-receptors (Witkin and Nelson, 2004; Sadek and Stark, 2015; Sadek et al., 2016c). Notably, H3R antagonists/inverse agonists have been found to exhibit a unique feature by their potential cognition-enhancing property as indicated by numerous lines of evidence from preclinical studies. Accordingly, several H3R antagonists/inverse agonists have been previously found to counteract DIZ-induced memory deficits in rodents (Witkin and Nelson, 2004; Passani and Blandina, 2011; Sadek and Stark, 2015; Sadek et al., 2016c) Furthermore, previous preclinical as well as clinical experiments revealed that antagonists at *N*-methyl-D-aspartate receptors (NMDARs), e.g., ketamine, promote cognitive deficits in healthy humans and exaggerate symptomatic parameters in patients with schizophrenia (Luby et al., 1959; Ghoneim et al., 1985; Javitt and Zukin, 1991; Krystal et al., 1994; Malhotra et al., 1997; Brown et al., 2013). NMDAR antagonists were, also, found to induce behavioral deficits in rodents through impairment of their neurocognitive functions (Large, 2007). In addition, there are several evidences that central histamine significantly alter cognitive deficits and that antagonists/inverse agonists selectively targeting central histamine H3Rs may possibly lead to therapeutic entities with potential clinical use in cognitive symptoms, e.g., AD (Witkin and Nelson, 2004; Esbenshade et al., 2008; von Coburg et al., 2009; Sadek and Stark, 2015; Sadek et al., 2016c). Notably, numerous developed H3R antagonists were revealed in their effects to decrease ketamine- and DIZ-induced cognitive deficits in several animal models of schizophrenia (Browman et al., 2004), signifying that these drugs may also be effective against CDS (Witkin and Nelson, 2004; Bardgett et al., 2010; Charlier et al., 2013; Sadek and Stark, 2015; Sadek et al., 2016c). Moreover, previous preclinical experiments showed that several H3R antagonists, e.g., ABT-239 and A-431404, significantly reduced ketamine- and DIZ-induced cognitive deficits in rats when compared to standard antipsychotics, e.g., olanzapine and risperidone (Brown et al., 2013). Based on the high attention level generated by these preclinical outcomes, the central H3Rs represent an attractive target for developing novel H3R antagonists/inverse agonists with the potential role in neuropsychiatric multi-neurotransmitter disorders, e.g., AD and CDS (Yokoyama et al., 1993; Yokoyama, 2001; Harada et al., 2004; Witkin and Nelson, 2004; Uma Devi et al., 2010; Bhowmik et al., 2012; Khan et al., 2015; Sadek and Stark, 2015; Sadek et al., 2016a,c). Therefore, the effects of the newly developed highly potent and selective non-imidazole H3R antagonist/inverse agonist, E159 [1-(6-(2,3-dihydro-1*H*-inden-5-yloxy)hexyl)-3-methylpiperidine], with high *in vitro* selectivity toward H3Rs (Lazewska et al., 2006) (**Figure 1**) has been investigated on its behavioral effects on DIZ-induced memory deficits in PAP and NOR tasks in adult male rats. Also and since anxiety and motor activity could confound learning and memory’s performance of animals (Sadek et al., 2016e), the effects of E159 on locomotor activity and anxiety-like behaviors of the same animals in EPM were tested. Moreover, the abrogative effects of PYR, ZOL, and SCO on the E159-provided memory-enhancing effects in PAP and NOR tests were assessed.

## Materials and Methods

### Animals

Inbred male Wistar rats aged 6–8 weeks (body weight: 180–220 g, Central Animal Facility of the UAE University) were maintained in an air-conditioned animal facility room with controlled temperature (24°C ± 2°C) and humidity (55% ± 15%) under a 12-h light/dark cycle. The animals were given free access to food and water. All experimental procedures were conducted between 9:00 and 14:00 h. The procedures used to assess effects of E159 were approved by the Institutional Animal Ethics Committee of CMHS/UAEU(A30-13). All efforts were considered to reduce number of animals used and their suffering. Also, all behavioral studies were conducted by the same experimenter.

### Drugs

RAMH dihydrochloride, H1R antagonist PYR, H2R antagonist ZOL dimaleate, DOZ hydrochloride, DIZ hydrogen maleate, and SCO hydrobromide were obtained from Sigma–Aldrich (St. Louis, MO, United States). Chemical synthesis, analysis, and approval of the structure for E159 [1-(6-(2,3-dihydro-1*H*-inden-5-yloxy)hexyl)-3-methylpiperidine] and PIT were conducted in the Department of Technology and Biotechnology of Drugs (Kraków, Poland) as described previously (Lazewska et al., 2006). DZP manufactured by Gulf Pharmaceutical Industries (Ras Al Khaimah, United Arab Emirates) was obtained from Dr. Ameen Al Amaydah (Department of Emergency Medicine, Emirates International Hospital, Al Ain, United Arab Emirates). All doses were expressed in terms of the free base of all drugs. The drugs used in the current study were dissolved in saline and injected i.p. at a volume of 1 ml/kg. All experimental procedures were carried out in a blinded fashion in which the experimenter was uninformed about the specific treatment groups to which an animal group belonged.

### Behavioral Tests

#### Step-Through PAP Test

**FIGURE 2:**
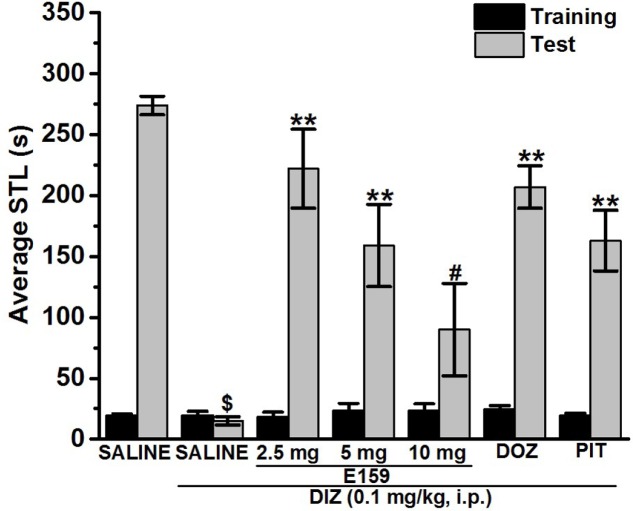
Effects of E159 on DIZ-induced memory deficits in an inhibitory avoidance conditioned response in rats. Gray columns represent the mean STLs measured during the retention test (test latencies) and black columns the mean STLs measured during the training trial before the delivery of the foot-shock (pre-shock latencies). Rats were injected with E159 (2.5, 5, or 10 mg/kg, i.p.), DOZ (1 mg/kg, i.p.), or PIT (10 mg/kg, i.p.) 30–45 min before the test session. ^∗∗^*P* < 0.001 for mean STLs vs. the value of the (saline)-treated group. ^#^*P* < 0.005 for mean STLs vs. the value of the [E159 (2.5 mg)]-treated group. ^$^*P* < 0.001 for mean STLs vs. the value of the (saline)-treated group. Data are expressed as mean ± SEM (*n* = 7).

**FIGURE 3:**
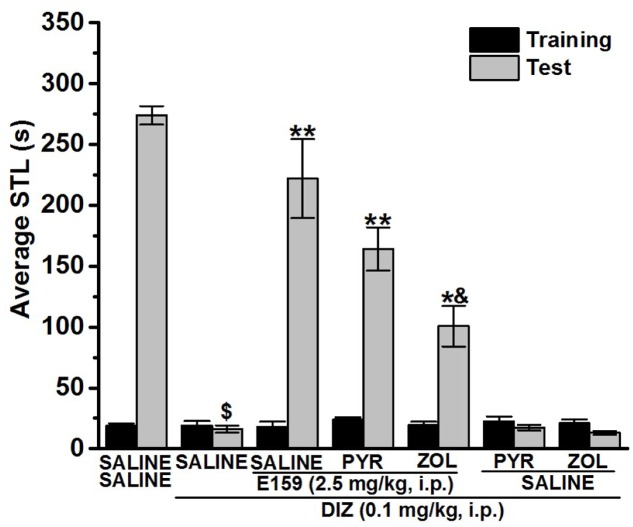
Effect of vehicle, E159, PYR, and ZOL on DIZ-induced deficit in an inhibitory avoidance conditioned response in rats. Gray columns represent the mean STLs measured during the retention test (test latencies) and black columns the mean STLs measured during the training trial before the delivery of the foot-shock (pre-shock latencies). Rats were injected with E159 (2.5 mg/kg, i.p.), PYR (10 mg/kg, i.p.) or ZOL (10 mg/kg, i.p.) 30–45 min before the test session. ^$^*P* < 0.001 for mean STLs vs. the value of the (Saline)-treated group. ^∗∗^*P* < 0.001 for mean STLs vs. the value of the (DIZ)-treated group. ^∗^*P* < 0.001 for mean STLs vs. the value of the (DIZ + E159)-treated group. ^&^*P* < 0.05 vs. the value of the (DIZ)-treated group. Data are expressed as mean ± SEM (*n* = 7).

**FIGURE 4:**
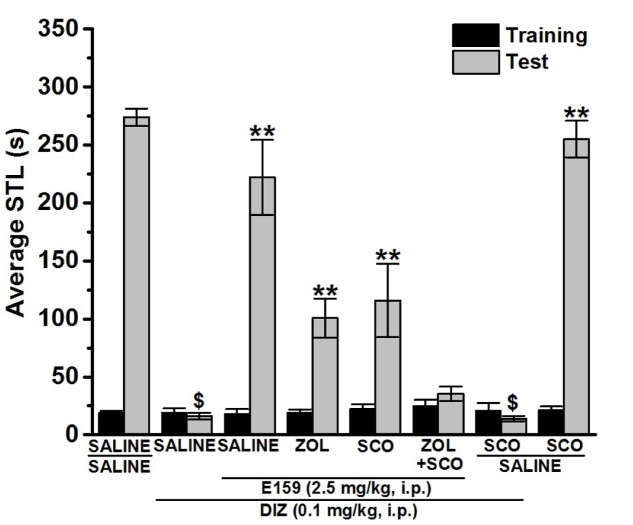
Effect of vehicle, E159, ZOL, and SCO on DIZ-induced deficit in an inhibitory avoidance conditioned response in rats. Gray columns represent the mean STLs measured during the retention test (test latencies) and black columns the mean STLs measured during the training trial before the delivery of the foot-shock (pre-shock latencies). Rats were injected with E159 (2.5 mg/kg, i.p.), ZOL (10 mg/kg, i.p.), SCO (1 mg/kg, i.p.) or a combination of ZOL (10 mg/kg) + SCO (1 mg/kg) 30–45 min before the test session. ^$^*P* < 0.001 for mean STLs vs. the value of the (Saline)-treated group. ^∗∗^*P* < 0.001 for mean STLs vs. the value of the (DIZ)-treated group. ^#^*P* < 0.05 for mean STLs vs. the value of the (DIZ + E159 + ZOL)- and (DIZ + E159 + SCO)-treated group vs. the value of the (DIZ)-treated group. Data are expressed as mean ± SEM (*n* = 7).

The step-through PAP test was done as previously described and in an automatically operated commercial Passive Avoidance Apparatus (step-through cage, 7550, Ugo Basile, Comerio, Italy) (Bernaerts et al., 2004; da Silva et al., 2009; Goshadrou et al., 2013; Khan et al., 2015; Sadek et al., 2015, 2016a,e). The experimental procedure consisted of two trials (training and testing) separated by a 24 h interval. Each rat in the first trial was placed in the white compartment, facing the auto guillotine door and, after a 30-s habituation period, the door was raised automatically, and cut-off time of 60 s was given for the animal to cross to the dark compartment. As soon as the rat placed all four paws in the dark module, the guillotine door was lowered and a scrambled foot-shock of 0.4 mA (20 Hz, 8.3 ms) was delivered to the grid floor for 3 s. Rats that failed to move within this period were excepted from the test on the following day. Directly after receiving the shock, the rat was removed from the dark chamber, returned to its home-cage, and the chambers were thoroughly cleaned. For the second and third training day, the same procedure was followed with the only change that a 300 s cut-off latency was allowed for the test animal to enter the dark compartment, however, without delivery of scrambled foot-shock. Animals that failed to cross into the dark compartment during the training, despite the practices conducted in training sessions, were excluded from the current study. For each experiment, 9–11 rats having the same average of age and weight were trained on the step-through latency (STL) test. Approximately 2–4 rats failed to show improved performance in a cut-off time of 60 s, a time period provided for the animals to cross to the dark compartment. In the current experiments, a sample group of seven rats was used for each STL experiment conducted for the PAP. In the test session, animals were turned amnesic with SCO (2 mg/kg) or DIZ (0.1 mg/kg) 30–45 min prior to the test session, and the rats were given a maximum of 300 s to move into the dark box. In this test session, the STL time taken by the animal to enter the dark box or STL in 5 min was recorded and documented. In order to identify a procognitive effect, 11 groups were acutely pretreated with Saline + Saline, DIZ (0.1 mg) + Saline, DIZ (0.1 mg) + E159 (2.5 mg), DIZ (0.1 mg) + E159 (5 mg), DIZ (0.1 mg) + E159 (10 mg), DIZ + DOZ (1 mg), or DIZ + PIT (10 mg) 30–45 min prior to the test session, respectively, and their effects on DIZ-induced memory deficits were measured by determining the STLs to enter the dark box. The E159-provided procognitive effect was confirmed by conducting additional experiments in which the respective promising dose of E159 (2.5 mg/kg) and PYR (10 mg/kg), ZOL (10 mg/kg), SCO (1 mg/kg), or a combination of SCO and ZOL were co-injected. The doses of SCO, PYR, and ZOL were selected according to previous studies (Orsetti et al., 2001, 2002; Khan et al., 2015; Sadek et al., 2015, 2016a,b,d) (**Figures 2**–**4**).

#### NOR Test

**FIGURE 5:**
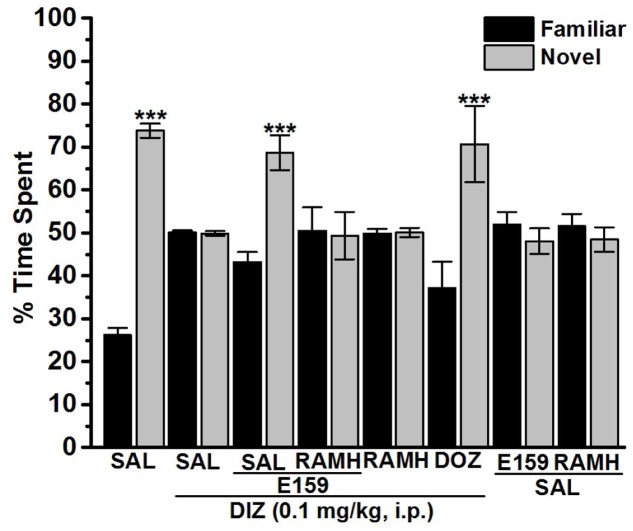
Effects of E159 on DIZ-induced STM cognitive deficits in the object recognition test in rats. Thirty minutes following training session T1, E159 (2.5 mg/kg), RAMH (10 mg/kg), DOZ (1 mg/kg), or DIZ (0.1 mg/kg) was administrated intraperitoneally. The test session T2 was performed 120 min (STM) after the training session T1. Results are calculated as individual percentage of time spent exploring familiar (black columns) and novel (gray columns) objects. Data represent mean ± SEM of 6–8 animals per experimental group. ^∗∗∗^*P* < 0.001 vs. respective familiar object.

**FIGURE 6:**
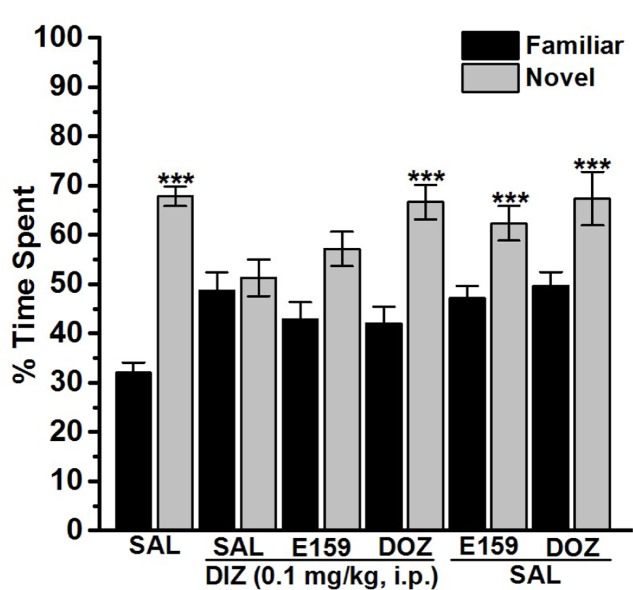
Effects of E159 on DIZ-induced LTM cognitive deficits in the object recognition test in rats. Thirty minutes following training session T1, E159 (2.5 mg/kg), DOZ (1 mg/kg), or DIZ (0.1 mg/kg) was administrated intraperitoneally. The test session T2 was performed 24 h (LTM) after the training session T1 in which E159 (2.5 mg/kg) was administered 30–45 min before T2. Results are calculated as individual percentage of time spent exploring familiar (black columns) and novel (gray columns) objects. Data represent mean ± SEM of 6–8 animals per experimental group. ^∗∗∗^*P* < 0.001 vs. respective familiar object.

Novel Object Recognition was assessed with a slight modification as previously described (Ennaceur and Delacour, 1988; Izquierdo et al., 1999; de Lima et al., 2005; Karasawa et al., 2008). The experiments were conducted in a black open field box measuring 50 cm × 35 cm × 50 cm. The experimental procedure included two sessions of habituation separated with a 1-h interval, whereby the animals were permitted for exploratory time of 3-min. On the test day, animals were placed in the test box, and after a 3-min of exploration, two objects (9 cm × 5 cm × 9 cm wood blocks which were in duplicate of the same size but different shape and color) were presented in two corners (approximately 30 cm apart from each other). The experimental session (24 h later) consisted of two trials, namely T1 and T2, each of a duration of 3 min. In T1, rats were exposed to two identical objects, and rats which explored the objects for less than 10 s during T1 were excluded from the experiments. In T2, performed 120 min for STM or 24 h for LTM later, animals were exposed to two objects, one of which was replaced with a new object and the other object was a duplicate of the familiar one to exclude olfactory traits. Also, the familiar or new object as well as the relative position of the two objects were counterbalanced and randomly permuted during trial T2. The measurement was obtained with the time spent by the animal exploring both objects during T1 and T2, and exploration of an object was defined as touching one of both objects with the nose. Other behavioral observations, e.g., turning around or sitting on the object was not considered an experimental behavior. The open field arena as well as the objects were carefully cleaned using 70% (volume/volume; v/v) alcohol. DIZ and all test compounds were dissolved in saline and administered i.p. 30 min following T1 at a volume of 1 ml/kg, and the doses were expressed in terms of the free base. The control groups received an equivalent volume of saline. The doses chosen for the NOR test were derived from the results in PAP and/or previously reported procognitive studies (Bernaerts et al., 2004; da Silva et al., 2009; Goshadrou et al., 2013; Khan et al., 2015; Sadek et al., 2015; Sultan et al., 2017). In order to detect a procognitive effect for STM, eight groups of 6–8 rats each were used. They were injected with Saline + Saline, DIZ + Saline, DIZ + E159 (2.5 mg/kg), DIZ + E159 (2.5 mg) + RAMH (10 mg), DIZ + DOZ (1 mg), DIZ + RAMH (10 mg), Saline + E159 (2.5 mg/kg), or Saline + RAMH (10 mg/kg) 30–45 min after T1, and their effects on DIZ-induced cognitive deficits (STM) were measured by determining the time spent by the rat in exploring objects during trials T1 and T2 (**Figure 4**). Moreover, the variable N-F/N + F which provides the discrimination index (*D*) was computed. Also and in order to exclude any confounding factors, E159 (2.5 mg/kg) and RAMH (10 mg/kg) were tested on their effect on two separate saline-treated control groups. The above mentioned experimental protocol was applied to detect the procognitive effect for LTM in another six groups of 6–8 rats each, however, with the only change that E159 (2.5 mg/kg, i.p.) was administered 30–45 min prior to T2. They were injected with Saline + Saline, DIZ + Saline, DIZ + E159 (2.5 mg/kg), DIZ + DOZ (1 mg), Saline + E159 (2.5 mg/kg), or Saline + DOZ(1 mg/kg) (**Figure 5**). In all experiments, doses of DIZ, RAMH, and DOZ were selected according to previous studies (Bernaerts et al., 2004; de Lima et al., 2005; da Silva et al., 2009; Goshadrou et al., 2013; Khan et al., 2015; Sadek et al., 2015, 2016a; Sultan et al., 2017) (**Figures 5**, **6**).

#### EPM Test

**FIGURE 7:**
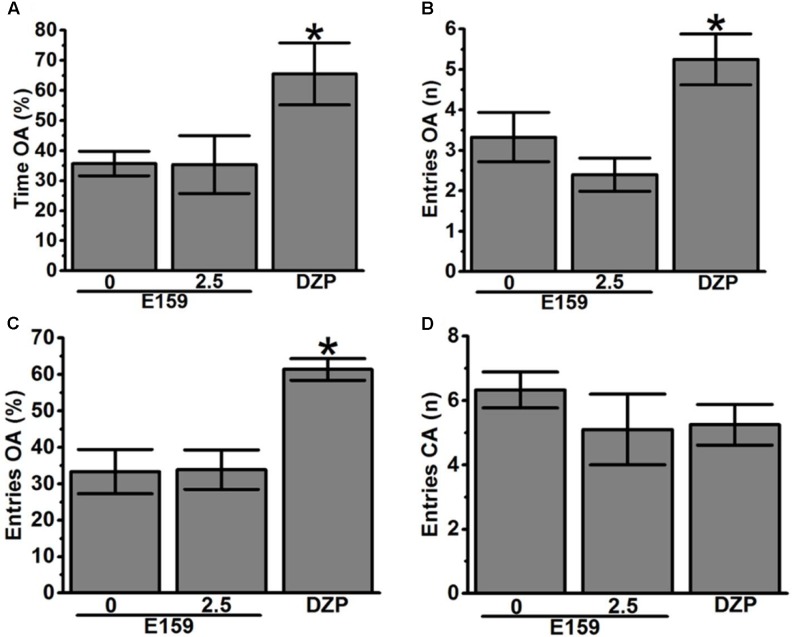
Effects of acute E159 pretreatment on exploratory behavior in the EPM test. E159 (0 or 2.5 mg/kg) did not change the percentage of time spent on the open arms of the EPM **(A)**, the number of entries into the open arms **(B)**, and the percentage of entries into the open arms **(C)**. Pretreatment with the H3R antagonist/inverse agonist E159 did not affect the number of closed arm entries **(D)**. However, DZP significantly increased the percentage of time spent on the open arms of the EPM **(A)**, increased the number of entries into the open arms **(B)** and the percentage of entries into the open arms **(C)**, without significant alteration in the number of closed arm entries **(D)**. Data are expressed as mean ± SEM (*n* = 8–10). ^∗^*P* < 0.05 for the value of DZP-treated group vs. the value of the E159 (Saline)- or (2.5 mg)-treated group.

Anxiety-like behaviors were with slight modification assessed in an EPM as previously described (Jiang et al., 2016). The EPM apparatus consisted of several parts including one central part (8 cm × 8 cm), two opposing open and closed arms (30 cm × 8 cm), and non-transparent walls (30 cm in height). The experiment room was light and temperature-controlled, and both the plat form and the wall were thoroughly cleaned between every session using 10% ethanol spray. Animals were placed individually in the center arena of the maze (50 cm above the floor) facing the open arm. The measurement was carried out by observing the amount of time spent with head and forepaws on the open arms and closed arms of the maze and the number of entries into each arm was manually scored for a duration of 5 min. In this experiment, the total number of entries into the closed arms is typically considered as an index for locomotor activity of the respective tested animal. In order to detect anxiety-like and locomotor behavior, three groups of 8–10 animals each were injected i.p. with saline (Saline group) and two test groups that received either E159 (2.5 mg/kg) or DZP (10 mg/kg) (**Figures 7A–D**).

#### Statistical Analysis

IBM^®^ SPSS Statistics^®^ version 20 software (IBM Middle East, Dubai, United Arab Emirates) was used for all statistical comparisons in all behavioral experiments. Data were expressed as means ± SEM. The effects of E159 on DIZ-induced memory deficits were analyzed using a two-way analysis of variance (ANOVA) with Treatment (vehicle or E159) and Dose (E159) as the between-subjects factor. The effect of E159 in combination with PYR, ZOL, or SCO on STL time was analyzed using one-way ANOVA with Treatment as the between-subject factor. The effects of E159 with the most promising dose in PAP (2.5 mg/kg) on DIZ-induced amnesia in NOR test were analyzed using a mixed repeated-measure two-way analysis of variance (ANOVA) with Treatment (vehicle or E159) and Dose (E159; 2.5 mg/kg, i.p.) as the between-subjects factor. The effect of E159 (2.5 mg/kg) in EPM test were assessed by measuring the time spent on the open arms and closed arms of the maze as well as the number of entries into each arm. The results observed in EPM test were analyzed using one-way ANOVA with Treatment as the between-subject factor. In case of a significant main effect, *post hoc* comparisons were performed with Bonferroni’s test. The criterion for statistical significance was set at *p* ≤ 0.05.

## Results

### Effects of E159, DOZ, and PIT on DIZ-Induced Memory Impairment in Step-Through PAP

**Figure 2** shows the effect of E159 (2.5, 5, and 10 mg/kg), DOZ (1 mg/kg), and PIT (10 mg/kg) on DIZ-induced memory impairments in step-through PAP in rats. When injected before the retention test, one-way analysis of variance showed that acute systemic prtreatment with E159 (2.5, 5, and 10 mg/kg), DOZ (1 mg/kg), and PIT (10 mg/kg) exihbited a significant effect on STLs [*F*_(13,84)_ = 9.691; *P* < 0.001]. Also and as compared to the (saline)-treated group, subsequent *post hoc* analyses showed that DIZ (0.1 mg/kg) decreased STL time with [*F*_(1,12)_ = 820.401; *P* < 0.001]. In addition, E159 (2.5 and 5 mg/kg), DOZ (1 mg/kg), and PIT (10 mg/kg) exerted significant memory-enhancing effect on STLs when compared to (DIZ)-treated group with [*F*_(1,12)_ = 34.631; *P* < 0.001], [*F*_(1,12)_ = 15.392; *P* < 0.001], [*F*_(1,12)_ = 95.365; *P* < 0.001], and [*F*_(1,12)_ = 30.074; *P* < 0.001], respectively. The procognitive effects observed for E159 at a dose of 2.5 mg/kg were not significantly different from (Saline)-treated rats (*p* = 0.1703). However, E159 tested at a dose of 10 mg/kg failed to exhibit significant memory enhancing effect when compared to (DIZ)-treated group (*p* = 0.093).

### Effects of PYR and ZOL on the E159-Provided Memory Improvement in DIZ-Induced Deficit in Step-Through PAP

For this experiment, separate groups of rats (*n* = 7 for each group) were pretreated with either the CNS-penetrant H1R antagonist PYR (10 mg/kg) or the CNS-penetrant H2R antagonist ZOL (10 mg/kg) 30–45 min prior to the test session. As shown in **Figure 3**, pairwise comparisons reported that, as expected, acute systemic pretreatment with E159 (2.5 mg/kg, i.p.) prolonged STL time when compared to the (DIZ)-treated group with [*F*_(1,12)_ = 34.346; *P* < 0.001]. This E159-provided prolongation of STL time was partly abrogated following ZOL [*F*_(1,12)_ = 9.437; *p* = 0.009: Saline + DIZ + E159 vs. DIZ + E159 + ZOL], but not following an acute systemic administration with PYR when compared to (Saline + DIZ + E159)-treated group [*F*_(1,12)_ = 2.082; *p* = 0.175]. Notably, neither Saline + Saline + DIZ vs. Saline + DIZ + PYR, nor Saline + Saline + Saline vs. Saline + DIZ + ZOL differences were found to be significant (*p* = 0.70 and *p* = 0.47, respectively) (**Figure 3**).

### Effects of ZOL, SCO, and a Combination of ZOL and SCO on the E159-Provided Memory-Improvement in DIZ-Induced Deficit in Step-Through PAP

As depicted in **Figure 4**, one-way analysis of variance showed that acute systemic pretest administration with E159 (2.5 mg/kg), ZOL (10 mg/kg), SCO (1 mg/kg), and combination of ZOL (10 mg/kg) with SCO (1 mg/kg) exerted a significant memory improving effect on STLs [*F*_(7,48)_ = 29.436; *P* < 0.001]. Moreover, subsequent *post hoc* analyses revealed that DIZ (0.1 mg/kg) reduced STL time with [*F*_(1,12)_ = 849.171; *P* < 0.001] when compared to the (Saline)-treated group (**Figure 4**). Furthermore, E159 (2.5 mg/kg) exhibited significant improving effect on STLs with [*F*_(1,12)_ = 34.346; *P* < 0.001] when compared to (DIZ)-treated group, and this E159-induced improvement of STL time was not completely abrogated following acute systemic co-administration with ZOL (10 mg/kg) or SCO (1 mg/kg) with [*F*_(1,12)_ = 21.382; *P* < 0.001: Saline + Saline + DIZ vs. DIZ + E159 + ZOL] and [*F*_(1,12)_ = 8.429; *P* < 0.05: Saline + Saline + DIZ vs. DIZ + E159 + SCO]. In addition, an acute systemic pretreatment with ZOL (10 mg/kg) combined with SCO (1 mg/kg) showed significantly higher abrogative effect on the E159-provided memory improvement when compared with the abrogation observed by ZOL or SCO administered alone with [*F*_(1,12)_ = 11.466; *P* < 0.05: DIZ + E159 + ZOL vs. DIZ + E159 + ZOL + SCO] and [*F*_(1,12)_ = 5.288; *P* < 0.05: DIZ + E159 + SCO vs. DIZ + E159 + ZOL + SCO], respectively. Interestingly, SCO failed to alter STL time in both (Saline)- and Saline + DIZ-treated group (*p* = 0.757 and *p* = 0.334, respectively) (**Figure 4**).

### Effects of E159 and DOZ on DIZ-Induced STM Deficits in NOR

The results observed in NOR test for the total time exploring both objects during training and test session of the respective group revealed that there were no significant differences between (Saline)- and DIZ-treated groups (**Table 1**). The latter observation is important to exclude any confounding factors, e.g., that the acute post-training injection with DIZ in the first experiment did not modulate locomotor activity or motivation as sensorimotor parameters. Also, statistical analyses of results observed for exploratory time during T1 revealed that no significant differences were present in exploration between the two identical objects. **Figure 5** shows the effect of E159 (2.5 mg/kg) and DOZ (1 mg/kg) on DIZ-induced STM deficits of memory in NOR. Moreover, one-way analysis of variance showed that acute systemic pretreatment with E159 (2.5 mg/kg) and DOZ (1 mg/kg) exhibited a significant effect on exploratory time spent with both objects in T2 with [*F*_(7,40)_ = 5.799; *P* < 0.001] when injected 30 min after training session T1. As shown by subsequent *post hoc* tests, DIZ (0.1 mg/kg) decreased memory for the novel object in T2 with [*F*_(1,12)_ = 205.423; *P* < 0.001] when compared to the (saline)-treated group. However, E159 (2.5 mg/kg) enhanced impaired STM when compared to (DIZ)-treated group with [*F*_(1,12)_ = 24.396; *P* < 0.001], and was comparable to the DOZ(1 mg/kg)-provided memory-enhancing effect (*p* = 0.706) (**Figure 5**). Moreover, discrimination indices measured for the different groups in STM support the latter observed results (**Table 1**).

**Table 1:** Effects of E159 on DIZ-induced total amount of time spent exploring both objects during object recognition training and test session in rats.

		Time exploring objects (s)				Discrimination index “*D*”^a^	
			
Group	*n*	STM T1	STM T2	LTM T1	LTM T2	STM	LTM
Saline	6	37.50 ± 3.20	37.83 ± 3.39	36.67 ± 2.95	39.83 ± 3.22	0.51 ± 0.05	0.36 ± 0.04
DIZ (0.1 mg/kg)	8	36.00 ± 2.73	42.25 ± 1.57	23.33 ± 2.01	22.67 ± 2.35	0.03 ± 0.01^∗^	0.03 ± 0.07^∗^
DIZ + E159 (2.5 mg/kg)	8	23.75 ± 2.18	22.67 ± 4.84	24.63 ± 2.74	27.67 ± 3.48	0.14 ± 0.05^#^	0.05 ± 0.09
DIZ + E159 (2.5 mg/kg) + RAMH (10 mg/kg)	6	37.50 ± 1.52	41.17 ± 2.43	ND	ND	0.06 ± 0.11	ND
DIZ + DOZ (1 mg/kg)	6	24.83 ± 1.72	25.33 ± 1.68	23.00 ± 3.34	21.00 ± 2.49	0.28 ± 0.11^∗^	0.32 ± 0.07^∗^
DIZ + RAMH (10 mg/kg)	6	23.50 ± 1.41	24.33 ± 1.59	ND	ND	0 ± 0.02	ND
Saline + E159 (2.5 mg/kg)	6	35.67 ± 2.93	37.33 ± 2.19	35.50 ± 2.78	36.83 ± 1.46	0.36 ± 0.07	0.24 ± 0.15
Saline + RAMH (10 mg/kg)	6	35.67 ± 2.93	32.33 ± 1.59	ND	ND	0.34 ± 0.09	ND


*Data are expressed as mean ± SEM of 6–10 animals per experimental group. There were no significant differences among training and test exploring time in STM and LTM group of each respective treatment.*

*^a^Discrimination index (*D*) calculated as; *D* = *N* -*F*/*N* + *F*, where *N* and *F* are the time spent with novel object and familiar object, respectively. ND, not determined. ^∗^*P* < 0.05 for mean *D* vs. the value of the (DIZ)-treated group vs. value of (Saline)-treated group. ^#^*P* < 0.05 for mean *D* vs. the value of the (Saline)- and (DIZ + E159)-treated group vs. the value of the (DIZ)-treated group. Data are expressed as mean ± SEM (*n* = 7).*

### Effects of E159 and DOZ on DIZ-Induced LTM Deficits in NOR

One-way analysis of variance revealed that acute systemic pretreatment with E159 (2.5 mg/kg) and DOZ (1 mg/kg) exerted no significant effect on time spent exploring objects in T2 with [*F*_(5,30)_ = 1.293; *p* = 0.293] when injected 30 min after training session T1 and 60 min before T2 and 24 h later (**Figure 6**). As shown by subsequent *post hoc* analyses, DIZ (0.1 mg/kg) impaired memory for the novel object in T2 with [*F*_(1,10)_ = 12.788; *P* < 0.05] when compared to the (Saline)-treated group (**Figure 6**). Moreover, acute systemic pretreatment with DOZ (1 mg/kg) enhanced LTM with [*F*_(1,10)_ = 7.485; *P* < 0.05] as compared to (DIZ)-treated group. However, E159 (2.5 mg/kg) failed to significantly enhance LTM when compared to (DIZ)-treated group with [*F*_(1,10)_ = 1.094; *p* = 0.320]. Also, acute systemic administration of E159 (2.5 mg/kg) or DOZ (1 mg/kg, i.p.) alone failed to modulate LTM in T2 when compared to the DIZ-treated group with [*F*_(1,10)_ = 2.285; *p* = 0.057] and [*F*_(1,10)_ = 0.765; *p* = 0.402], respectively (**Figure 6**). Also, observed discrimination indices for the different treated groups support the latter results in LTM (**Table 1**).

### Effect of E159 on Rat Performance in EPM Test

**Figure 7** shows the effects of acute administration of E159 (0 or 2.5 mg/kg) on the percentage of time spent in open arms, number of entries into open arms, percentage entries into open arms, and locomotor activity (number of entries into closed arm) of rats observed in the EPM test. *Post hoc* analyses indicated that compared to saline, E159 failed to alter the percentage of time spent exploring the open arms of the maze during a 5-min session with [*F*_(1,14)_ = 0.001, *p* = 0.981] as compared to the (Saline)-treated group (**Figure 7A**). Moreover, statistical analyses of data describing the number and percentage of entries into the open arms of the maze [*F*_(1,12)_ = 1.389, *p* = 0.261; *F*_(1,10)_ = 0.003, *p* = 0.954, respectively) generated essentially the same results. As shown in **Figures 7B,C**, no significant difference from that obtained with the (Saline)-treated group were observed following acute systemic administration with E159 (2.5 mg/kg). However, the percentage time spent in open arms and number and percentage of entries into open arms were significantly modulated after acute systemic administration of DZP (10 mg/kg, i.p.) with [*F*_(1,12)_ = 14.482, *P* < 0.05], [*F*_(1,10)_ = 13.257, *P* < 0.05], and [*F*_(1,11)_ = 15.805, *P* < 0.05], respectively (**Figures 7A–C**). Interestingly, the number of closed arm entries following E159 (2.5 mg/kg) or DZP (10 mg/kg) injections with [*F*_(1,14)_ = 0.554, *p* = 0.469] and [*F*_(1,12)_ = 1.311, *p* = 0.275] were not significantly changed, indicating that locomotor activity *per se* was not modulated subsequent to acute administrations of E159 or DZP when compared to that obtained with saline pretreatment (**Figure 7D**). Therefore, the detected behavioral alterations were not influenced by any substantial modifications in the traveled distance during the test period.

## Discussion

Mounting evidences show that acute systemic administration of NMDAR antagonists such as DIZ (Luby et al., 1959) reduces performance of experimental rodents in a wide-ranging varieties of learning and memory tasks (Javitt and Zukin, 1991) including PAP and NOR (Luby et al., 1959; Ghoneim et al., 1985; Javitt and Zukin, 1991; Krystal et al., 1994; Malhotra et al., 1997; Brown et al., 2013; Khan et al., 2015; Sadek and Stark, 2015; Sadek et al., 2016a,c). Therefore, DIZ-induced dementia has been commonly used to evaluate potential therapeutic agents for treating AD and CDS (Luby et al., 1959; Ghoneim et al., 1985; Javitt and Zukin, 1991; Krystal et al., 1994; Malhotra et al., 1997; Brown et al., 2013). In this study, acute systemic administration of E159 only at lower doses (2.5 and 5 mg/kg) significantly reversed the DIZ-induced memory deficits in PAP test in adult rats (**Figure 2**). It has been proposed that NMDA receptors participate with a significant function in several stages of memory, namely consolidation and retrieval processes (Vorobjev et al., 1993; Brabant et al., 2009, 2013). Therefore, it is possible that in our experiments E159 moderately reduced DIZ-induced memory deficits through direct stimulation of NMDA receptors by the increased release of central histamine as a consequence of blocking histamine H3 auto-receptors by this class of H3R antagonists. These results are in consensus with earlier observations in which histamine was found to improve transmission in cultured hippocampal cells mediated by NMDA receptors, indicating that the interaction between histamine and NMDA receptors possibly will enable the histamine’s capability reduce DIZ-induced memory deficits (Vorobjev et al., 1993; Xu et al., 2005; Brabant et al., 2013; Sadek et al., 2016a). Importantly, the memory-enhancing effect observed for E159 was dose-dependent, since the improvement of memory provided by E159 (2.5 mg/kg) in the DIZ-induced amnesia model was significantly higher when compared to the higher doses (5 and 10 mg/kg), demonstrating that an optimum in memory-enhancing effect was observed when the H3R antagonist/inverse agonist E159 was applied at the lowest dose (2.5 mg/kg), and an off-target effect for E159 at higher doses (5 and 10 mg/kg) might have been observed in the current study (**Figure 2**). The latter observations of dose dependency are, also, in agreement with earlier experimental results conducted in different rodents (Benetti and Izquierdo, 2013; Benetti et al., 2013; Sadek et al., 2015). Moreover, the observed cognitive enhancing effects for E159 (2.5 mg/kg) were similar to those obtained for the reference H3R antagonist/inverse agonist PIT and the reference drug DOZ (**Figure 2**). Furthermore, the E159 (2.5 mg/kg)-provided memory-enhancing effects were moderately reversed when rats were administered with the CNS-penetrant H2R antagonist ZOL but not with the CNS-penetrant H1R antagonist PYR. The latter observation confirmed our previous results observed for the non-imidazole H3R antagonist DL77 (2.5–10 mg/kg) in different memory processes, namely acquisition, consolidation, and retrieval (Sadek et al., 2016e). The ameliorative effects found for E159 in DIZ-induced memory deficits further indicate that histaminergic pathways through activation of H2Rs are fundamentally contributing in neuronal pathways important for alteration of retrieval processes. An additional experiment revealed that the E159-provided memory-enhancing effect was, also, moderately abrogated when animals were administered with SCO, however, significantly further abrogated when animals were administered with a combination of SCO and ZOL (**Figure 3**). The latter experimental finding clearly indicates that cholinergic muscarinic neurotransmission as well as histaminergic circuits through activation of H2Rs are strongly involved in the E159-provided memory enhancing effects (**Figure 3**). Unlike the PAP test, NOR test in rodents measures natural behavior of rodents and advantages from their distinctive curiosity for discovering their surroundings, and it does not comprise a punishment or a reward. Notably, the NOR is a paradigm used in rodent models to capture characteristics of the neurodevelopmental basis of CDS by interpreting the results without confounding factors, e.g., side effects such as antinociceptive effect of several old-generation imidazole-based H3R antagonists/inverse agonists, e.g., thioperamide (Jaaro-Peled, 2009; Tseng et al., 2009; Brown et al., 2013), and it was found to show high sensitivity to both cognition impairing (Ennaceur and Delacour, 1988; Ennaceur and Meliani, 1992a,b) and enhancing agents (Lebrun et al., 2000; Barak and Weiner, 2011). The results observed in the current study showed that acute systemic post-training administration of E159 (2.5 mg/kg) significantly improved the time spent to explore the novel object compared with the familiar object, and delivered a type of STM (**Figure 5**). These results are in line with earlier observations which revealed that numerous imidazole-based H3R antagonists, e.g., thioperamide and clobenpropit (Giovannini et al., 1999), and non-imidazole based H3R antagonists, e.g., PIT (Ligneau et al., 2007); GSK189254 (Giannoni et al., 2010), SAR110894 (Griebel et al., 2012), and ABT-239 (Provensi et al., 2016) ameliorate the amnesic effects of SCO, DIZ or time in rodents in NOR tests. Interestingly, the STM-enhancing effects provided with E159 in DIZ-induced memory deficits were significantly abrogated after animals were co-injected with the CNS-penetrant H3R agonist RAMH (**Figure 5**). The latter observations are in line with an earlier preclinical study in which RAMH abolished the memory-enhancing effects obtained by the imidazole-based H3R antagonist ciproxifan on STM in mice (Pascoli et al., 2009). Contrary, acute systemic post-training administration of E159 (2.5 mg/kg) failed to increase the time spent exploring the novel objects when compared with the familiar objects in LTM (**Figure 6**). These results in NOR obviously indicate that histaminergic H3Rs are profoundly contributing in neuronal circuits involved in the E159-provided STM-memory enhancing effects, but not in LTM-enhancing effects (**Figure 6**). Moreover, the discrepancies observed for E159 in PAP and NOR might be explained with the differences of conducts and measured features of both models in rodents. Accordingly, NOR measures natural behavior of animals and advantages from their distinctive curiosity for discovering their surroundings, and it does not comprise a punishment or a reward. Also, NOR is a commonly used paradigm in rodents capture characteristics of the neurodevelopmental basis of CDS (Jaaro-Peled, 2009; Tseng et al., 2009; Brown et al., 2013). Interestingly, several H3R antagonists were in previous preclinical studies identified as promising targets for CDS and were proposed to be of potential therapeutic value based on the fact that H3R functions as auto- and hetero-receptor, thus, modulating the biosynthesis and release of several neurotransmitters, including histamine, dopamine, and acetylcholine, which are important for cognitive functions (Brioni et al., 2011; Sadek and Stark, 2015; Sadek et al., 2016c). Notably, E159 at the dose of 2.5 mg/kg, the dose that provided the most promising procognitive effect in PAP and NOR, failed to change anxiety levels and locomotor activity of the tested rodents (**Figures 7A–D**). Moreover, E159 used at the same dose did not alter the number of closed arm entries, indicating that E159 did not change locomotor activity of rodents (**Figure 7D**). The latter observations are significant, since improved performance in PAP or NOR can be the consequence of several variables not related to memory-enhancing effects such as modifications in emotional responding or in spontaneous locomotor activity (McGaugh and Roozendaal, 2009; Charlier et al., 2013). These results are, also, in agreement with previous observations in which acute systemic administration of the H3R antagonist DL77 (2.5–10 mg/kg) failed to modify spontaneous locomotor activity of the same animal species in the open field test (Sadek et al., 2016e).

## Conclusion

The observed results show that the non-imidazole H3R antagonist E159 reduces DIZ-induced cognitive deficits in PAP and NOR task in adult male rats. Also, the results observed in PAP reveals that acute systemic pretreatment with E159 in DIZ-induced amnesia models significantly ameliorates cognitive impairments via mechanisms dependent on cholinergic muscarinic neurotransmission and – at least partially – H2Rs activation. Moreover, the present results strongly support the potential therapeutic value of histamine H3R antagonists in the treatment of neuropsychiatric diseases, e.g., AD and CDS. Nonetheless, additional preclinical experiments are still warranted with a series of further behavioral test models and with different rodent species to increase the validity of the translational value for possible applicability of H3R antagonists/inverse agonists in the modulation of memory impairment in several neuropsychiatric diseases.

## Author Contributions

BS was responsible for the study concept, design, acquisition, and analysis of animal data. AA conducted behavioral experiments. KK-K and DŁ were responsible for the generation, synthesis, and pharmacological *in vitro* characterization the H3R antagonist DL77. BS drafted the manuscript. KK-K, DŁ, and AA provided critical revision for the manuscript. All authors critically reviewed content and approved final version for publication.

## Conflict of Interest Statement

The authors declare that the research was conducted in the absence of any commercial or financial relationships that could be construed as a potential conflict of interest.

## Abbreviations

AD: Alzheimer disease
CDS: cognitive deficits associated with schizophrenia
DIZ: dizocilpine
DOZ: donepezil
DZP: diazepam
EPM: elevated plus maze
H3Rs: histamine H3 receptors
LTM: long-term memory
NOR: novel object recognition
PAP: passive avoidance paradigm
PIT: pitolisant
PYR: pyrilamine
RAMH: *R*-(α)-methyl-histamine
SCO: scopolamine
STL: step-through latency
STM: short-term memory
ZOL: zolantidine
